# Liquor Acidi Phosphorici

**Published:** 1881-08

**Authors:** 


LIQUOR ACIDI PHOSPHORICI (without iron) and LIQUOR ACIDI PHOSPHORICI COMPOUND (with iron).—The formulæ of the above-named preparations were devised by Professor Pepper, of Philadelphia, as a substitute for an empirical preparation known as "Acid Phosphate," and largely advertised in the newspapers.

Our experience with the several preparations of phosphates of various names has been considerable within a few years past, and quite satisfactory in some instances. Still there seemed to be something of that class of preparations needed as a tonic to the digestive organs, and to the nervous system in the exhaustion resulting often from long-continuous study and overwork, which none of the usual preparations supplied.

Our attention having been called to the above articles, as prepared by Messrs. John Wyeth & Bro.,of Philadelphia, in accordance with Dr. Pepper's formulæ ; we tried them personally, and have prescribed them with entire satisfaction for others. We feel satisfied that they are applicable to a wider range of troubles than we have used them for thus far, and would confidently recommend them to the profession.

